# Ferroptosis is involved in PGPS-induced otitis media in C57BL/6 mice

**DOI:** 10.1038/s41420-022-01025-1

**Published:** 2022-04-21

**Authors:** Bin Yan, Daoli Xie, Yuancheng Wu, Shuli Wang, Xiaolin Zhang, Tong Zhao, Luying Liu, Peng Ma, Guqiang Li, Ying Yang, Yucheng Zhao, Tihua Zheng, Ruishuang Geng, Bo Li, Qingyin Zheng

**Affiliations:** 1 Hearing and Speech Rehabilitation Institute, College of Special Education, Binzhou Medical University, Yantai, China; 2 Rehabilitation Medicine & Physical Therapy, School of Rehabilitation Medicine, Binzhou Medical University, Yantai, China; 3 Department of Otolaryngology/Head and Neck Surgery, Institute of Otolaryngology, Affiliated Hospital of Binzhou Medical University, Binzhou, China; 4 Department of Pathology, School of Basic Medicine, Binzhou Medical University, Yantai, China; 5 Department of Genetics, School of Basic Medicine, Binzhou Medical University, Yantai, China; 6 Department of Otolaryngology-Head & Neck Surgery, Case Western Reserve University, Cleveland, OH USA

**Keywords:** Acute inflammation, Diseases

## Abstract

Otitis media (OM) is a common disease that can cause hearing loss in children. Currently, the main clinical treatment for OM is antibiotics, but the overuse of antibiotics might lead to bacterial resistance, which is a worldwide public health challenge. Studying the pathogenesis of OM will help us develop new effective treatments. Ferroptosis is one type of programmed cell death characterized by the occurrence of lipid peroxidation driven by iron ions. Many studies have shown that ferroptosis is associated with infectious diseases. It is presently unclear whether ferroptosis is involved in the pathogenesis of OM. In this study, we explored the relationship between ferroptosis and OM by PGPS-induced OM in C57BL/6 mice and treating the induced OM with ferroptosis inhibitors deferoxamine (DFO), Ferrostatin-1 (Fer-1), and Liperoxstatin-1 (Lip-1). We examined the expression of ferroptosis-related proteins acyl-CoA synthetase long chain family member 4 (ACSL4) and prostaglandin-endoperoxide synthase 2 (Cox2), glutathione peroxidase 4 (GPX4) protein as well as lipid peroxidation markers 4-hydroxynonenal (4-HNE) and malondialdehyde (MDA). The results showed that in PGPS-induced OM model mice, several ferroptosis-related proteins including ACSL4 and Cox2 were up-regulated compared to mice treated with saline. Meanwhile, a ferroptosis-related protein GPX4 was down-regulated upon PGPS treatment. The DFO treatment in PGPS-inoculated mice effectively inhibited the development of OM. The inhibitors treatment caused a significant decrease in the expression of ACSL4, Cox2, 4 HNE, MDA, reduction in free iron. Meanwhile, the ferroptosis inhibitors treatment caused increase in the expression of inflammation-related factors tumor necrosis factor-α (TNF-α) and antioxidant protein GPX4. Our results suggest that there is a crosstalk between ferroptosis signaling pathway and the pathogenesis of OM. Ferroptosis inhibition can alleviate PGPS-induced OM.

## Introduction

Otitis media (OM) is a common disease that many children younger than 3 years of age will experience [1]. Common complications of OM include conductive hearing loss, tympanic membrane, perforation, and mastoiditis [2]. If treatment is not in time, OM will cause long-term or permanent hearing loss [3]. Currently, the common treatment of OM is the application of antibiotics [4]. However antibiotic resistance is a major public health challenge. A better understanding of the mechanism underlying OM pathogenesis would help to develop a new effective treatment.

Ferroptosis is a type of iron-dependent programmed cell death, characterized by the occurrence of lipid peroxidation [5]. Ferroptosis is characterized morphologically by increased mitochondrial membrane density [6]. Iron is an essential trace element that participates in many important functions, including oxygen transport, DNA synthesis, metabolic energy, and cellular respiration, through oxidation-reduction reactions [7, 8]. Iron catalyzes the production of large amounts of reactive oxygen species (ROS) by participating in the Fenton reaction and can lead to cell death [9, 10]. ROS accumulates mainly through the acyl-CoA synthetase long chain family member 4 (ACSL4) pathways. Increased expression of ACSL4 leads to the oxidation of polyunsaturated fatty acids [11, 12]. Reduced activity of glutathione peroxidase 4 (GPX4) which is another important cause of lipid peroxidation in cells [13, 14]. As one of the most important antioxidant enzymes, GPX4 directly reduces lipid peroxides and prevents cell damage [15, 16].

Due to the strong redox ability of iron, iron overload could cause many problems. Iron imbalance is related to many diseases, including tuberculosis [17], Alzheimer’s disease [18], and Parkinson’s disease [19]. Several reports have shown a correlation between iron homeostasis and inflammation, and iron overload could lead to the incidence of inflammatory diseases [20–22]. Researchers have demonstrated that excess iron leads to lipid peroxidation of cell membranes and causes disruption of polyunsaturated fatty acid metabolism [23]. Polyunsaturated fatty acids such as AA are released from cell phospholipid membranes and converted into prostaglandins through prostaglandin-endoperoxide synthase 2 (Cox2), which activates immune cells to induce inflammation [14, 24, 25]. Many studies have shown Cox2 as a downstream molecular marker of ferroptosis. Interestingly, Cox2 has been shown to be involved in OM [26]. Therefore, we speculate that by regulating the expression of Cox2, the ferroptosis pathway is inhibited, thereby reducing the symptoms of OM.

The common ferroptosis inhibitors are deferoxamine (DFO), Ferrostatin-1 (Fer-1), and Liperoxstatin-1 (Lip-1), researchers in several studies have investigated the beneficial effects of ferroptosis inhibitor on inflammation. For example, researchers have reported that DFO as a ferroptosis inhibitor significantly relieves inflammation [27]. It is reported that Lip-1, another ferroptosis inhibitor, decreased the release of interleukin 6 (IL-6), interleukin-1beta (IL-1β), and tumor necrosis factor-α (TNF-α) [28]. Many studies also showed that Fer-1 alleviates inflammatory responses caused by Lipopolysaccharides (LPS). Fer-1 prevented LPS-induced upregulation of TNF-α, IL-1β, and IL-6 protein expression [29]. DFO inhibits the onset of cellular ferroptosis by chelating intracellular iron ions. DFO has been commonly used to treat acute and chronic iron overload in patients with thalassemia who has undergone long-term blood transfusion [30]. DFO is a safe and effective iron-chelating agent and has been widely used in thalassemia clinical treatment for more than 20 years [31, 32]. Therefore, we chose DFO to treat OM and to further investigate the role of ferroptosis pathway in the development of OM. We also used Lip-1 and Fer-1, to observe their therapeutic effects on OM (Figs. S1 and S2).

**Fig. 1:**
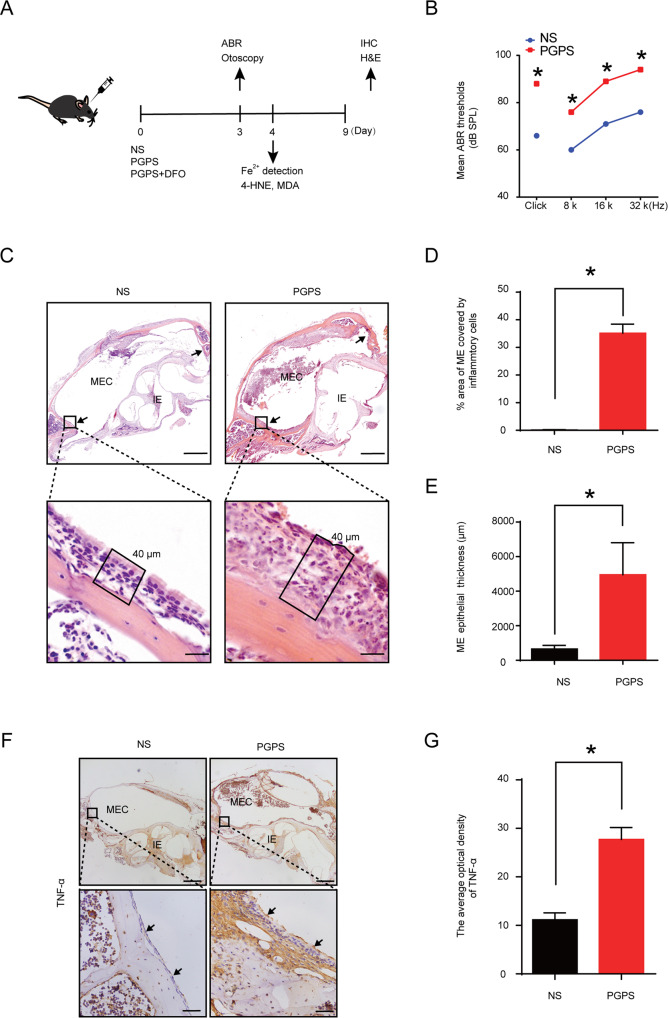
PGPS could induce OM in mice. **A** Workflow of the experiments. B6 mice were divided into three groups, the only NS group, the only PGPS group, and the PGPS and DFO co-injection group. Three days after injecting the mice with the drug, we tested the ABR thresholds and performed an otoscope observation. On the fourth day, the mice were collected and observed tissue iron and lipid peroxidation products. H&E staining and immunohistochemical were performed on the ninth day. **B** The ABR threshold results showed that the hearing threshold of the PGPS group was significantly higher than that of the NS group. **C** Compared the H&E staining of the PGPS group and the NS group, the histology showed inflammations in the middle ear cavity (MEC) in PGPS group. The inner ear (IE) was absent of inflammations. **D** Statistically analyzed the thickness of the middle ear epithelium and **E** the inflammatory cells in the middle ear cavity. In the PGPS group, the middle ear epithelial hyperplasia was obvious, and inflammatory cell infiltration appeared in the middle ear cavity. The upper scale bar: 500 μm. The lower scale bar: 20 μm **F** Immunohistochemical analysis of TNF-α. **G** Compared with the NS group, the expression of TNF-α in the MEC of the PGPS group was significantly higher. The upper scale bar: 500 μm. The lower scale bar: 50 μm. The data are shown as mean ± SD. *P < 0.05, (PGPS group: *n* = 5, NS group: *n* = 5).

Our research purpose is to study the possible crosstalk between the role of ferroptosis in OM and the therapeutic effect of the ferroptosis inhibitor DFO in OM mice. The experiment was carried out in accordance with the experimental workflow (Fig. 1A). This study suggests that ferroptosis plays a pathogenic role in OM and that the inhibition of ferroptosis could reduce the inflammatory response in OM.

## Results

### DFO treatment improves hearing function and middle-ear morphology in B6 mice with PGPS-induced OM

OM mouse model was developed as previously described by injecting PGPS [33]. Three days later, the mice are assessed for OM. The hearing threshold of mice with OM was significantly increased (Fig. 1B). Hematoxylin-Eosin (H&E) staining showed that the mice in the PGPS group had a stronger inflammatory response than those in the NS group (Fig. 1C). The middle ear inflammatory secretions increased (Fig. 1D), and the epithelium in the PGPS group significantly thickened (Fig. 1E). The expression of TNF-α in the PGPS group significantly increased (Fig. 1F, G).

**Fig. 2:**
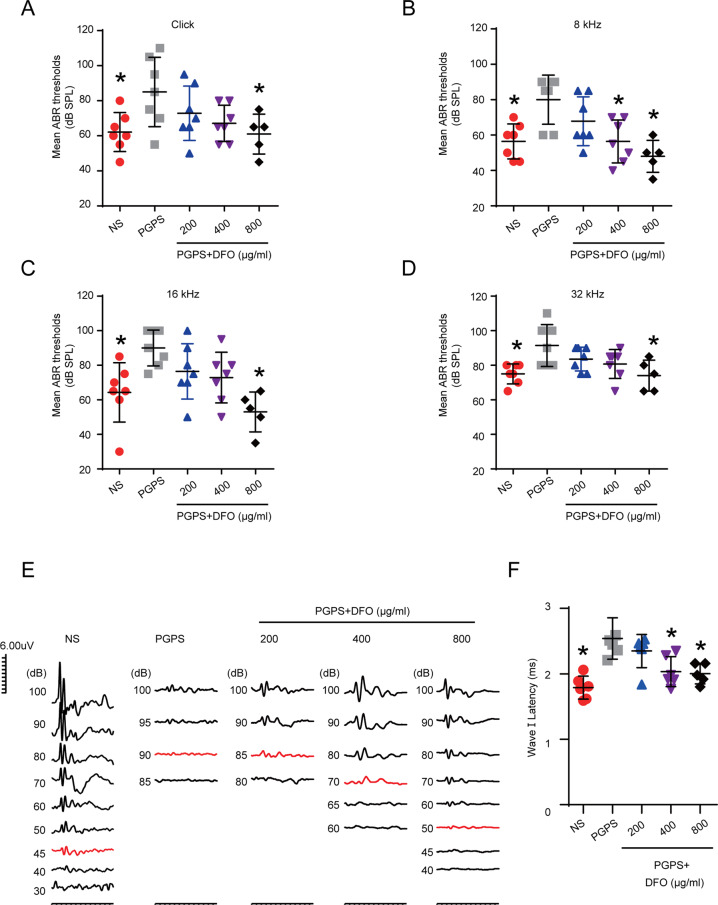
DFO improves the ABR threshold in mice with PGPS-induced OM. Comparison of the average ABR thresholds of mice in the PGPS, saline, and DFO groups for clicks of (**A**), 8 kHz (**B**), 16 kHz (**C**), and 32 kHz (**D**). The 400 μg/ml and 800 μg/ml DFO groups of 8, 16, and 32 kHz are statistically significant. (PGPS group: *n* = 7, NS group: *n* = 7, 200 μg/ml DFO group: *n* = 7, 400 μg/ml DFO group: *n* = 7, 800 μg/ml DFO group: *n* = 5). **E** Comparison of the ABR waveform of mice in the PGPS, saline, and DFO groups. **F** And the latency of a wave I with a sound intensity of 100 dB at 8 kHz frequency is calculated, (PGPS group: *n* = 6, NS group: *n* = 6, 200 μg/ml DFO group: *n* = 6, 400 μg/ml DFO group: *n* = 6, 800 μg/ml DFO group: *n* = 5). The data are shown as mean ± SD. *P < 0.05.

**Fig. 3:**
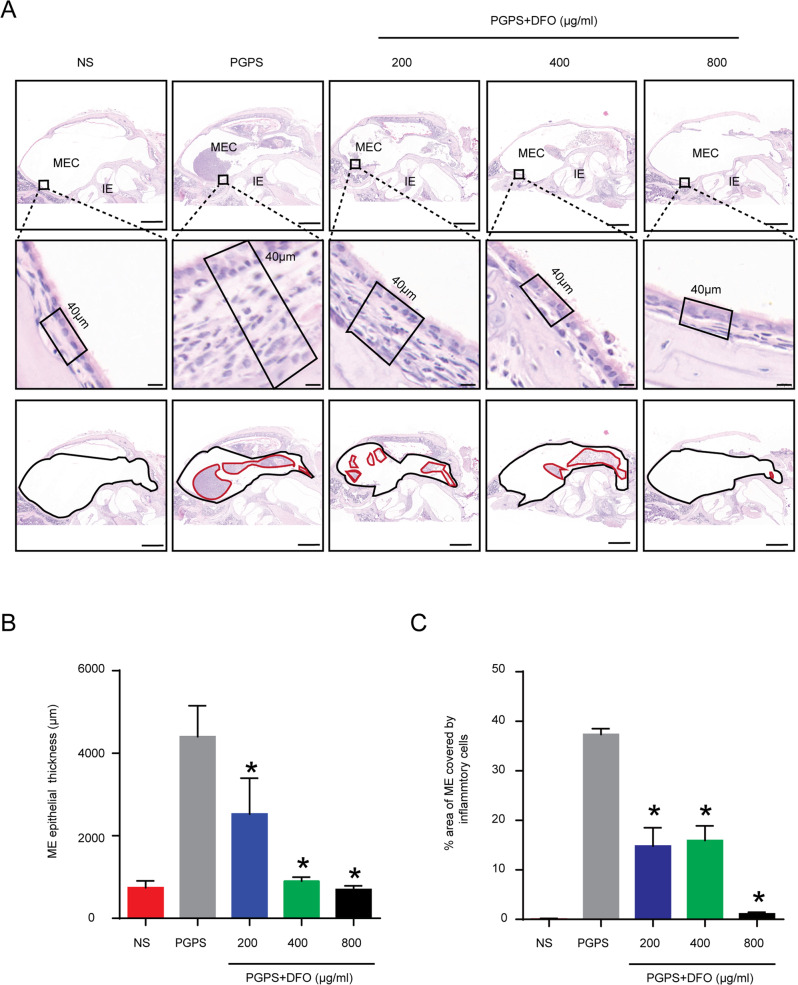
Hematoxylin-Eosin (H&E) staining shows the histological changes in middle ear epithelium after PGPS injection and various doses of DFO treatment. The mice are randomly divided into NS, PGPS, and DFO-treatment groups. Mice in the DFO-treatment group received treatments of three different concentrations, 200 μg/ml, 400 μg/ml, and 800 μg/ml. **A** The results of HE staining showed the degree of inflammation in the MEC of the five groups of mice. The IE was absent of inflammations. Scale bar: 500 μm. **B** The epithelial thickness in the middle ear cavity. Scale bar: 20 μm. **C** The inflammatory cells areas in the middle ear cavity. Scale bar: 500 μm. Inflammatory cells area and middle ear epithelial thickness were analyzed with ImageJ. The data are shown as mean ± SD. *P < 0.05, *n* = 3.

We injected three different concentrations of DFO to test its effect on OM and tested the hearing threshold of mice by ABR three days later. The ABR results showed that in the 800 μg/ml DFO group, the ABR thresholds for click frequencies (Fig. 2A) and pure tone frequencies of 8 kHz (Fig. 2B), 16 kHz (Fig. 2C), and 32 kHz (Fig. 2D) were all significantly decreased compared with the PGPS group. The wave I latency under 100 dB decibel sound stimulation in the 800 μg/ml DFO group was significantly shorter (Fig. 2E, F). There were no statistically significant differences in ABR thresholds in another two treatment groups compared to the PGPS group. Histological examination clearly showed that DFO treatment reduced inflammation in the middle ear of B6 mice (Fig. 3A). We choose 20 μM Lip-1 and 60 μM Fer-1 to intervene in PGPS-induced OM based on pre-experimental result. The ABR results showed that the thresholds of Lip-1 and Fer-1 groups were significantly lower at click frequencies (Fig. S1A), 8k (Fig. S1B), 16k (Fig. S1C), and 32k (Fig. S1D), which indicated that these two ferroptosis inhibitors could also significantly improve the hearing loss. Compared with the PGPS group, the three DFO treatment groups showed significantly reduced middle ear epithelial hyperplasia (Fig. 3B) and secretions (Fig. 3C). The epithelial hyperplasia and secretions in the 800 μg/ml DFO treatment group was also decreased compared to other doses of the DFO treatment group. The DFO 800 μg/ml treatment group achieved good therapeutic results both in hearing function and histology. Therefore, we chose 800 μg/ml of DFO as the treatment dose in the following experiments. We also observed the effect of Lip-1 and Fer-1on the OM of mice by H&E staining (Fig. S1E). The results showed that middle ear epithelial hyperplasia (Fig. S1F) and secretions (Fig. S1G) reduced compared with PGPS group.

### Inhibition of ferroptosis reduced inflammatory response of OM

**Fig. 4:**
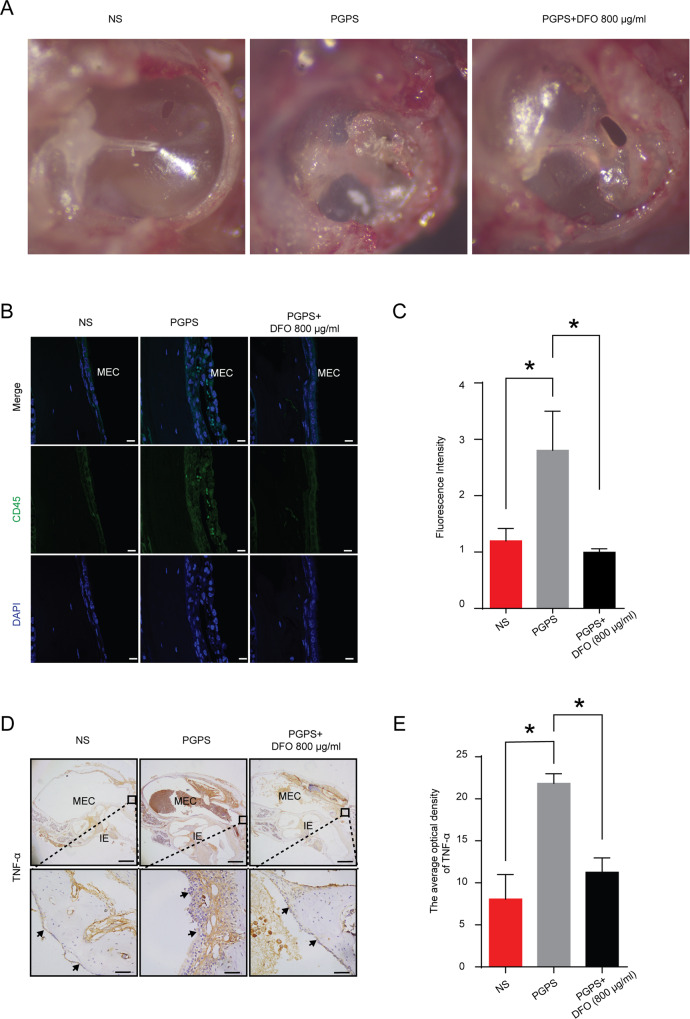
DFO reduces the OM response induced by PGPS. **A** After injection of PGPS, the presence of pus and tympanic membrane hyperplasia was clear, but the DFO-treatment group showed significantly less pus and a smaller degree of tympanic membrane hyperplasia than the PGPS group. **B** Immunofluorescence staining with CD45 (green) and DAPI (blue) is shown. Scale bars: 10 μm. **C** Relative fluorescent intensity of CD45. We analyzed the fluorescence intensity with ImageJ. **D** Immunohistochemical analysis of TNF-α (**E**) and analysis of the relative amount of TNF-α in the MEC. The fluorescent intensity is analyzed with ImageJ. *P < 0.05. scale bars: 10 μm. The upper scale bar: 500 μm. The lower scale bar: 50 μm. The data are shown as mean ± SD. *P < 0.05, *n* = 3.

We examined the tympanic membranes. Compared with those of the NS group, the tympanic membranes of the mice injected with the PGPS group showed obvious thickening and exudation of inflammatory substances. The tympanic membrane of the mice injected with 800 μg/ml of DFO also showed a certain degree of thickening in the tympanic membrane. However, the degree of proliferation and the inflammatory exudation decreased compared with the PGPS injected group (Fig. 4A). Then, we examined leukocyte infiltration by fluorescent light staining of the CD45, which is a pan leukocyte marker [34], and found that the PGPS group was obvious compared with the NS group, the leukocyte infiltration in the DFO-treatment group significantly reduced compared to the PGPS group (Fig. 4B, C). The expression of TNF-α (Fig. 4D, E) in the DFO-treatment group decreased. In summary, these results demonstrated that DFO had therapeutic effect on OM.

### DFO regulates the levels of lipid peroxidation and iron in OM and improves mitochondrial damage caused by inflammation

**Fig. 5:**
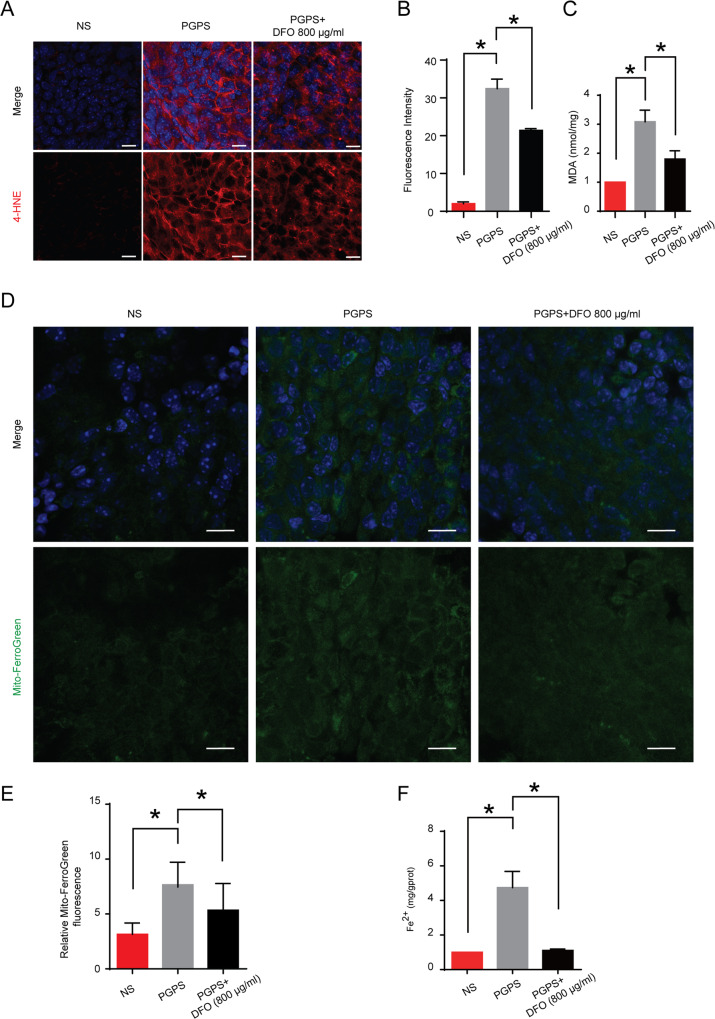
The effect of DFO on lipid peroxidation and iron accumulation in middle ear epithelial tissue of OM. **A** Immunofluorescence staining with 4-HNE. Scale bar: 10 μm. **B** Relative fluorescent fluorescent light intensity of 4-HNE. **C** Activity of amounts of MDA. The activity of 4-HNE and MDA is analyzed with ImageJ. Compared with the NS group, the expression of 4-HNE and MDA in the epithelial tissue of the middle ear in the PGPS group increased. The expressions of 4-HNE and MDA were lower in the DFO-treatment group and the PGPS group than in the PGPS group. **D** Relative fluorescent fluorescent light intensity of Mito-FerroGreen. Scale bar: 10 μm. **E** Relative fluorescent fluorescent intensity statistics of Mito-FerroGreen. **F** Activity of amounts of iron. The activity of iron is analyzed with ImageJ. Compared to the NS group, the expression of iron in the epithelial tissue of the middle ear in the PGPS group increased. The expressions of iron were lower in the DFO-treatment group and the NS group than in the PGPS group. The data are shown as mean ± SD. *P < 0.05, *n* = 3.

**Fig. 6:**
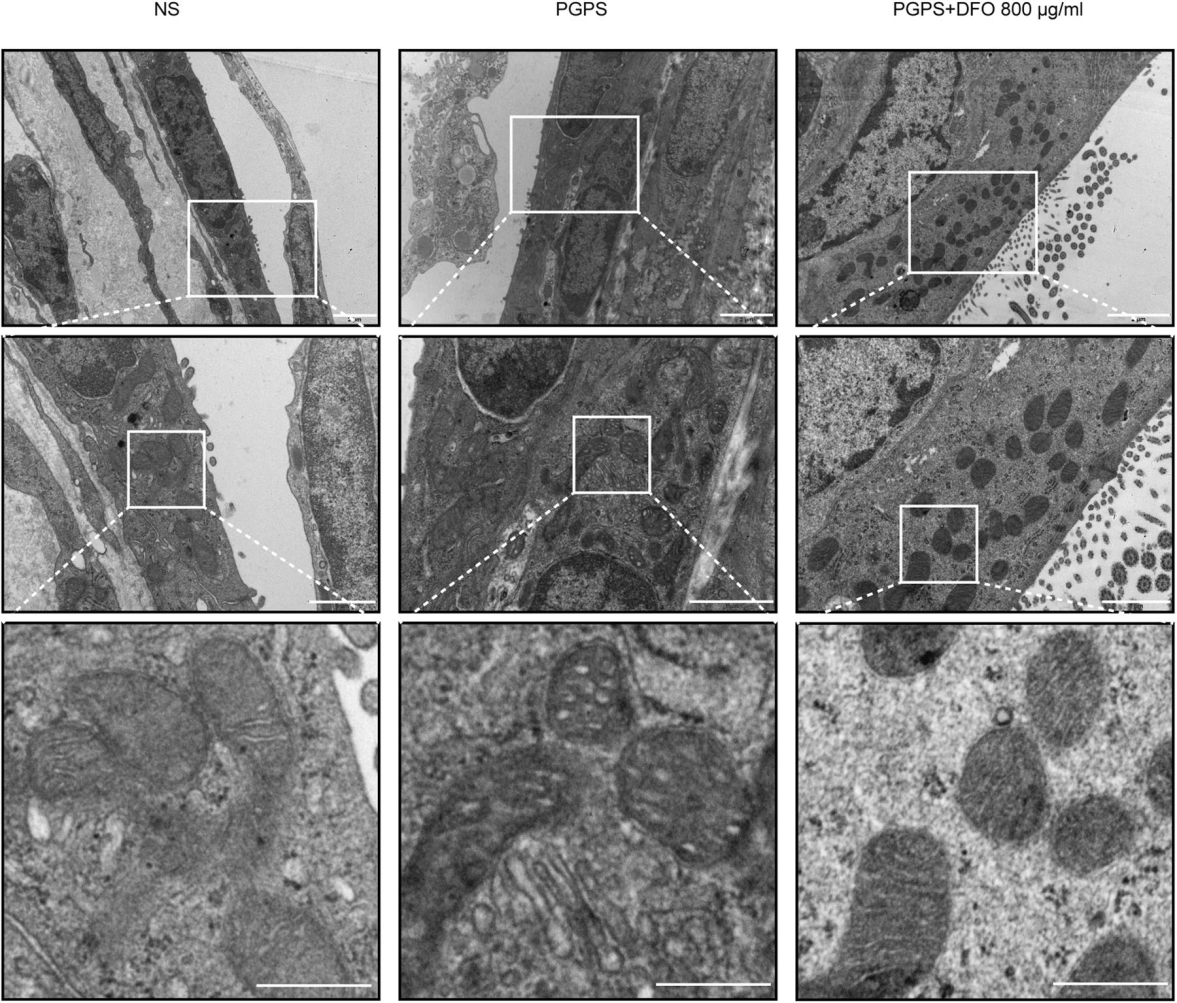
DFO attenuates inflammation-induced mitochondrial damage. Transmission electron microscopy images of middle ear epithelial tissue treatment of NS, PGPS, or PGPS + 800 μg/ml DFO. Scale bars: upper, 2 µm; middle, 1 µm; lower, 500 nm, *n* = 3.

4-HNE is an indicator of lipid peroxidation, the increase in 4-HNE is accompanied by an increase in malondialdehyde (MDA), implicating lipid peroxidation. We found that 4-HNE and MDA were upregulated in the PGPS group compared with that in the NS group. After DFO administration, 4-HNE (Fig. 5A, B) and MDA (Fig. 5C) were inhibited in the DFO treatment group. Fer-1 and Lip-1 inhibit ferroptosis by inhibiting the lipid peroxidation [35], so we checked the MDA content in middle ear tissue. The results showed that MDA levels were also significantly reduced after Lip-1 and Fer-1 injections (Fig. S2C). Next, we used Mito-FerroGreen and tissue iron kits to detect the accumulation of iron in mitochondria and cytoplasm. In the epithelial tissue of the middle ear of mice in the PGPS group, iron levels were increased in the cytoplasm and mitochondria (Fig. 5D–F). After DFO injection, the iron content in mitochondria and cytoplasm significantly decreased. Ferroptosis causes cellular mitochondrial membranes to crumple while mitochondrial cristae are reduced. In PGPS-induced middle ear epithelial cells of mice with OM, we observed many smaller mitochondria compared to the NS group, while the mitochondrial membranes of these mitochondria were crinkled. After DFO treatment, the mitochondria did not differ significantly from the NS group (Fig. 6).

### DFO regulates the level of ferroptosis-related proteins

**Fig. 7:**
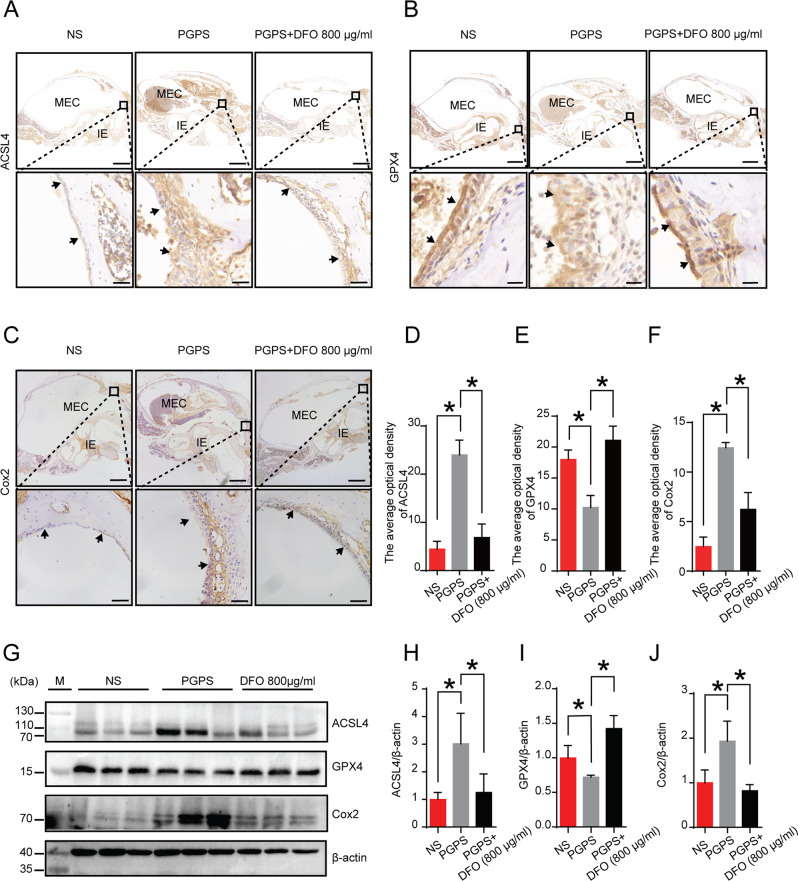
Inflammation affects the expression of ferroptosis-related proteins. **A**–**C** Immunohistochemical analysis of ACSL4, GPX4, Cox2, and **D**–**F** analysis of the relative amount of ACSL4 and GPX4 in the MEC. The quantitative analysis is analyzed with ImageJ. The upper scale bar: 500 μm. The lower scale bar: 50 μm. The data are shown as mean ± SD. *P < 0.05, *n* = 3. **G** Western blot analysis of GPX4, ACSL4, and Cox2. **H**–**J** Quantification of WB intensities. The data are shown as mean ± SD. *P < 0.05, *n* = 3.

GPX4 prevents ferroptosis through clearance of lipid peroxides. Thus, we examined the expression of GPX4 by Immunochemistry (IHC) and Western Blot (WB) (Fig. 7A, G). PGPS treatment significantly suppressed GPX4 protein expression compared with normal mice. GPX4 protein expression was increased significantly after treatment with DFO (Fig. 7D, H). According to previous research, ACSL4 could reduce inflammation by reducing the production of arachidonic acid (AA) derived pro-inflammatory lipid mediators. Thus, we investigated whether DFO regulated the expression of ACSL4 (Fig. 7B, G). The IHC and WB results showed that the expression of ACSL4 was increased in middle ear epithelial tissues in the PGPS group. The protein level of ACSL4 was up-regulated upon DFO application (Fig. 7E, I). The expression of a downstream molecular marker of ferroptosis (Fig. 7C, G) Cox2 was also significantly increased in the PGPS group. DFO treatment decreased the expression of Cox2 (Fig. 7F, J). Taken together, DFO may decrease ACSL4 expression in inflammation. These results indicate that DFO alleviates PGPS-induced OM via inhibiting the expression of ACSL4 and promoting the expression of GPX4. The expression of *ACSL4* and *Cox2* were decreased by Lip-1 and Fer-1. There are three inhibitors can regulate the gene expression of ferroptosis.

## Discussion

Previous studies have shown that various gene defects and endoplasmic reticulum stress are significantly related to OM [36–38]. However, the mechanism of OM development is still not fully understood. In recent years, some studies have shown that ferroptosis is a common cause in the pathogenesis of many diseases [39], there is no report on the relationship between ferroptosis and OM. Previous studies have shown that the expression of Cox2 is up-regulated in the OM middle ear epithelium but not in the normal middle ear of BALB/c mice [26]. In the present study, we found that the middle ear epithelial tissues of PGPS group mice increased the expression of Cox2 protein through the ferroptosis pathway, which promoted the inflammatory response. DFO could effectively reduce the level of iron in tissues, alleviate lipid peroxidation, inhibit ferroptosis and play an anti-inflammatory role at the same time.

Ferroptosis and inflammation have a two-way relationship. Some inflammatory factors directly affect the expression of ferroptosis-related proteins, and the occurrence of ferroptosis will promote inflammation. In previous studies, researchers found that the treatment of aortic endothelial cells in mice with IL-6 interrupted the iron homeostasis in cells, leading to ferroptosis of the cell [40]. They reported that ferroptosis promotes the expression of inflammatory factors when cancer [41, 42] and other diseases occurs [43, 44]. Previous studies have also shown that iron overload could lead to an increase in inflammatory diseases [45, 46]. In our study, we found that when PGPS-induced inflammation occurred in the middle ears of B6 mice, the expression of ACSL4 and Cox2 both was increased and the expression of GPX4 at the middle ear epithelial cells was decreased. This finding is consistent with that of Yanhong Shou et al. [47], who also found the changes in ferroptosis-related protein expression during inflammation.

We explored the mechanism of OM by using three ferroptosis inhibitors. DFO treatment has been shown to influence the expressions of GPX4 and ACSL4 [11, 48]. This is consistent with our finding that DFO can inhibit the expression of ACSL4 and promote the expression of GPX4. Under the joint regulation of ACSL4 and GPX4 proteins, unsaturated fatty acids act as substrates for lipid peroxidation and control the formation of 4-HNE and MDA [49, 50]. Lipid peroxidation increases the expression of Cox2 protein, which induces inflammation. The entry of DFO into cells inhibited the Fenton response leading to upregulation of GPX4 and downregulation of ACSL4. Lipid peroxidation was inhibited and Cox2 expression was decreased, suppressing ferroptosis and inflammation. Lip-1 and Fer-1 had the same therapeutic effect in OM as DFO. After treatment with Lip-1 and Fer-1, the MDA content in middle ear tissue was reduced. Meanwhile, we found that the expression of *ACSL4* and *Cox2* were consistent with the results after treatment with DFO by qRT-PCR. However, the specific mechanism of action still needs to be continued to be investigated.

**Fig. 8:**
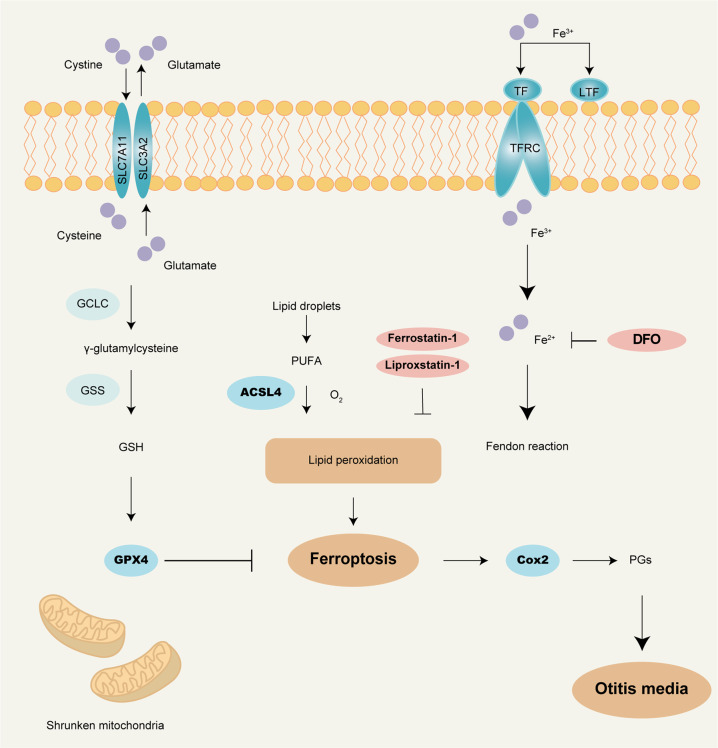
The general mechanism of ferroptosis. After Fe^3+^ enters the cell membrane through transferrin-1, it is converted into Fe^2+^. After a series of processes in the Fenton reaction, it finally affects the expression of GPX4 and ACSL4, leading to lipid peroxidation and ferroptosis. The arachidonic acid the oxidation reaction produces subsequently produces prostaglandins under the action of the Cox2, finally leading to inflammation. DFO is a chelating agent of iron. After DFO enters the cells, it causes the reduction of iron, thereby inhibiting the Fenton reaction and ultimately resulting in up-regulation of GPX4 and down-regulation of ACSL4, the inhibition of lipid peroxidation, and the inhibition of ferroptosis and inflammation. Lip-1 and Fer-1 are produced by direct inhibition of cellular lipid peroxidation, which leads to inhibition of ferroptosis and ultimately inhibition of inflammation.

The schematic diagram shows the mechanism underlying ferroptosis as OM (Fig. 8). Our study demonstrates the involvement of the ferroptosis signaling pathway in the pathogenesis of OM in the mouse model. The important role of iron ions in the pathogenesis of OM could provide a new therapeutic target for the treatment of OM. DFO could have a positive therapeutic effect on OM. However, the mechanism of the regulation of ACSL4 and GPX4 expression by DFO is currently unclear. We speculate that the protein changes may be related to the Fenton reaction, but how iron regulates the expression of proteins still needs further research. In summary, elevated levels of iron ions in the tissues lead to abnormal expression of GPX4 and ACSL4 proteins. Through the regulation of Cox2 protein, the level of iron promotes the occurrence of inflammation. Inhibition of iron ions in cells can effectively control inflammation. Our research findings also point to the possibility of developing effective treatment of other inflammatory diseases via intervention of ferroptosis signaling pathway.

## Methods

### Animals

C57BL/6 mice were purchased from the Nanjing model animal research center (Nanjing, China) and housed in a temperature and humidity controlled specific-pathogen-free animal facility. A total of 67 C57BL/6 mice, 8 weeks old, were used in this study, including 34 female mice and 33 male mice. The Animal Use and Care Committee of Binzhou Medical University approved the experimental protocol. Animals were randomly distributed over the different experimental conditions and blinding was applied during data acquisition and analysis when possible. OM was induced by PGPS as previously reported, the dose of PGPS was 5.5 mg/ml [51]. They were treated for normal saline, PGPS (BD Bioscience, San Jose, USA), and PGPS + DFO (MCE, USA). We divided the mice into five groups: the control group (NS group), OM model group (PGPS group), PGPS + 200 μg/ml DFO (200 μg/ml DFO group), PGPS + 400 μg/ml DFO (400 μg/ml DFO group), and PGPS + 800 μg/ml DFO treatment group (800 μg/ml DFO group). The drug is injected through the tympanic membrane into the middle ear cavity using a Hamilton syringe. After three days, the inflammatory effect of PGPS and the therapeutic effect of DFO was detected. After determining the appropriate dose of DFO treatment, the following experimental mice were equally divided into three groups: the normal saline group, the PGPS group, and the DFO treatment group (800 μg/ml). To further verify the relationship between ferroptosis and OM, we used two another ferroptosis inhibitors, Lip-1, and Fer-1. First, we performed a pre-experiment to determine the optimal drug concentration of lip-1 20 μM and Fer-1 60 μM. Mice were randomly divided into five groups, NS, PGPS, DMSO, Fer-1, and Lip-1 group to perform the next experiments.

### The auditory brainstem response (ABR) and the otoscopic examination

The ABR Thresholds were assessed before and at 3 days post-injection with PGPS [33]. The mice were anesthetized with 4% chloral hydrate and maintained their body temperature at 37 °C. ABR evoked by click and pure-tone 8, 16, and 32 kHz bursts. After the ABR tests, otoscopy was performed on drug-injected ears to determine the condition of inflammation.

### Hematoxylin-Eosin (H&E) staining of the middle ear

All mice were sacrificed by cervical dislocation, and the bullae (including both the middle and inner ear) were isolated and dissected. The bullae were immersed in 4% paraformaldehyde (PFA), and fixation took for 24 h at 4 °C. Then the tissues were decalcified with 10% EDTA solution for 3 days, and embedded in paraffin. The tissue sections (5 μm) were stained with hematoxylin-eosin and were observed under an optical microscope (Leica DMI4000 B, Germany).

### Immunofluorescence (IF) & Immunochemistry (IHC)

As described previously [52], the tissue sections were deparaffinized in xylene and gradient alcohol (100, 95, 85, 75, 50%, and triple distilled water), then treated with or incubated with sodium citrate buffer (pH6.0). After being washed three times in PBS for 5 min each time, the tissue sections were blocked in 5% bovine serum albumin for 1 h and were incubated with primary antibodies at 4 °C overnight. The primary antibodies used in this study are CD45 (IF: 1:200, Abcam, ab10558), 4-HNE (IF: 1:25, Abcam, ab48506), TNF-α (IHC: 1:100, China, Beijing, Proteintech, 60291-1-Ig), Cox2 (IHC: 1:100, USA, Massachusetts, Cell Signaling Technology, #12282), ACSL4 (IHC: 1;100, Abcam, ab155282) and GPX4 (IHC: 1;100, Abcam, ab125006). After three washes in 1 x PBS for 5 min each time and subsequent incubation with the secondary antibody (IF: CD45, goat anti-rabbit, 1:500, 4-HNE, goat anti-mouse, 1;500; IHC: ZSGB-BIO, SP-9000) for 1 h at room temperature. For samples stained with IF, tissue samples were counterstained with DAPI (4′,6-diamidino-2-phenylindole) for 5 min after secondary antibody application. For samples stained with IHC, tissue samples were counterstained with hematoxylin, dehydrated, and mounted. Fluorescence images were acquired under 40 × objective lens using Zeiss microscope (Zeiss, LSM880, Germany). IHC images were acquired under 40 × objective lens using Leica microscope (Leica DMI4000 B, Germany).

### Mitochondrial Fe^2+^ detection

To detect Fe^2+^ in middle ear epithelial cells, we used Mito-FerroGreen (Dojindo, Japan, M489) according to the manufacturer’s protocol. On the third day of PGPS inoculation, the right ear blister of the mouse was removed, and the middle ear epithelial cells were removed and placed in a petri dish. Add 1 mol/L Mito-FerroGreen working solution and incubate in a 37 °C, 5% CO_2_ incubator. Finally, the cells were observed under a fluorescent microscope (Zeiss, LSM880, Germany).

### Tissue Fe^2+^ and malondialdehyde (MDA) detection

The Tissue Fe^2+^ and MDA levels were detected by tissue iron assay kit (Jiancheng, Nanjing, A039-2-1), tissue MDA assay kit (Jiancheng, Nanjing, A003-1-2) according to the manufacturers’ protocols. The relative levels were analyzed by measuring the OD value.

### Transmission electron microscopy (TEM)

After pre-cooling the fixative and the container, the obtained middle ear mucosal tissue was cut into small pieces of 2 mm × 2 mm × 1 mm within 1 min. Then it was immersed in 2.5% glutaraldehyde fixative solution, fixed for three hours, rinsed with 0.2 mmol/L phosphate buffer (pH 7.2), fixed with 2% osmium tetroxide for 2 h, and then subjected to 0.2 mmol/L rinse with phosphate buffer (pH 7.2). Next, gradient dehydration was performed. 30, 50, and 70% alcohol was dehydrated for 8 min, and 90% alcohol was dehydrated for 12 min. Then, the dehydrating agent and the embedding agent were mixed and dehydrated one to one for one hour, the dehydrating agent and the embedding agent were mixed one to two for dehydration for 2 h, and finally the pure embedding agent was dehydrated for 5 h. The specimen was transferred to a plastic capsule at 70 °C and embedded in Epon812 epoxy resin after 10 h. Perform ultra-thin sectioning, with an area of about 0.2–0.3 mm^2^, stained with uranyl acetate and led sodium citrate, placed a wax plate in a clean petri dish, dropped the uranyl acetate dye solution on the wax plate, and removed the sliced copper. The net was covered on the dye solution for 30 min, then rinsed with three-distilled water and blotted dry. Finally, covered the copper mesh on the lead citrate dye solution for 10 min, then rinsed with double distilled water, blotted it dry, and observed under the electron microscope.

### Western blot

The bullae tissues were lysed with (Radio Immunoprecipitation Assay) RIPA lysis and an extraction buffer (ThermoFisher Scientific, 89900). Equal amounts of proteins (60 μg) were subjected to dodecyl sulfate, sodium salt-Polyacrylamide gel electrophoresis (SDS-PAGE), and transferred onto a polyvinylidene difluoride (PVDF) membrane. The membranes were blocked with 5% nonfat-dried milk and then probed overnight at 4 °C with relevant primary antibodies: anti-ACSL4 (Proteintech, 22401-1-AP), anti-GPX4 (Abcam, ab125006), anti-Cox2 (Proteintech, 12375-1-AP) and anti-β-actin (CST, mAb #8457). After washing with Tris-Buffered Saline-Tween 20 (TBS-T), membranes were probed again for 1 h at room temperature with a species-specific secondary antibody (Abcam, Ab6721) coupled with horseradish peroxidase. The western blot bands were detected using a Chemiluminescent HRP Substrate kit (Merck Millipore, WBKLS0100) and visualized using a ChemiDoc XRS + System (Bio-Rad, Hercules, CA, USA). The intensities of protein bands were measured and quantified using ImageJ software from the National Institutes for Health.

### Statistical analysis

Each group of mice was randomly assigned and each experiment was repeated at least three times. No samples or animals were excluded from the analysis. GraphPad Prism 9 software was used for statistical analysis. Data are expressed as mean ± SD. Unpaired Student’s *t* test was used to determine statistical significance when comparing two groups, and one-way analysis of variance (ANOVA) were used when comparing more than two groups. *P* values less than 0.05 were considered significant.

## Supplementary information

Supplemental data

supplementary material

## Data Availability

Data are available to the journal and the publisher upon request.
