# Supplementary material for: Ferroptosis is involved in PGPS-induced otitis media in C57BL/6 mice
Source: Cell Death Discov. 2022 Apr 21;8:217. doi: 10.1038/s41420-022-01025-1 (PMC9023543; doi:10.1038/s41420-022-01025-1)
Supplement: Supplementary file 1 — Supplemental data [file 41420_2022_1025_MOESM1_ESM.docx]

**Supplemental data**

**Supplemental methods**

**Quantitative real-time polymerase chain reaction (qRT-PCR)**

Total RNA was isolated from frozen OM tissues using TRIzol Reagent (Invitrogen, USA). The quality and concentrations of RNA were measured using a NanoDrop 2000c spectrophotometer (Thermo Scientific, USA). With 1 μg of RNA in the 20 μL reaction system, the total RNA was reverse transcribed into cDNA using the PrimeScriptTM RT reagent kit (TaKaRa, China) according to the manufacturer's instructions. Quantitative realtime PCR was performed with SYBR Green Master (Roche, Switzerland) in a multicolor real-time PCR detection system (Bio-Rad iQ5, USA). The primer sequences in this study are listed in Supplementary Table 1.


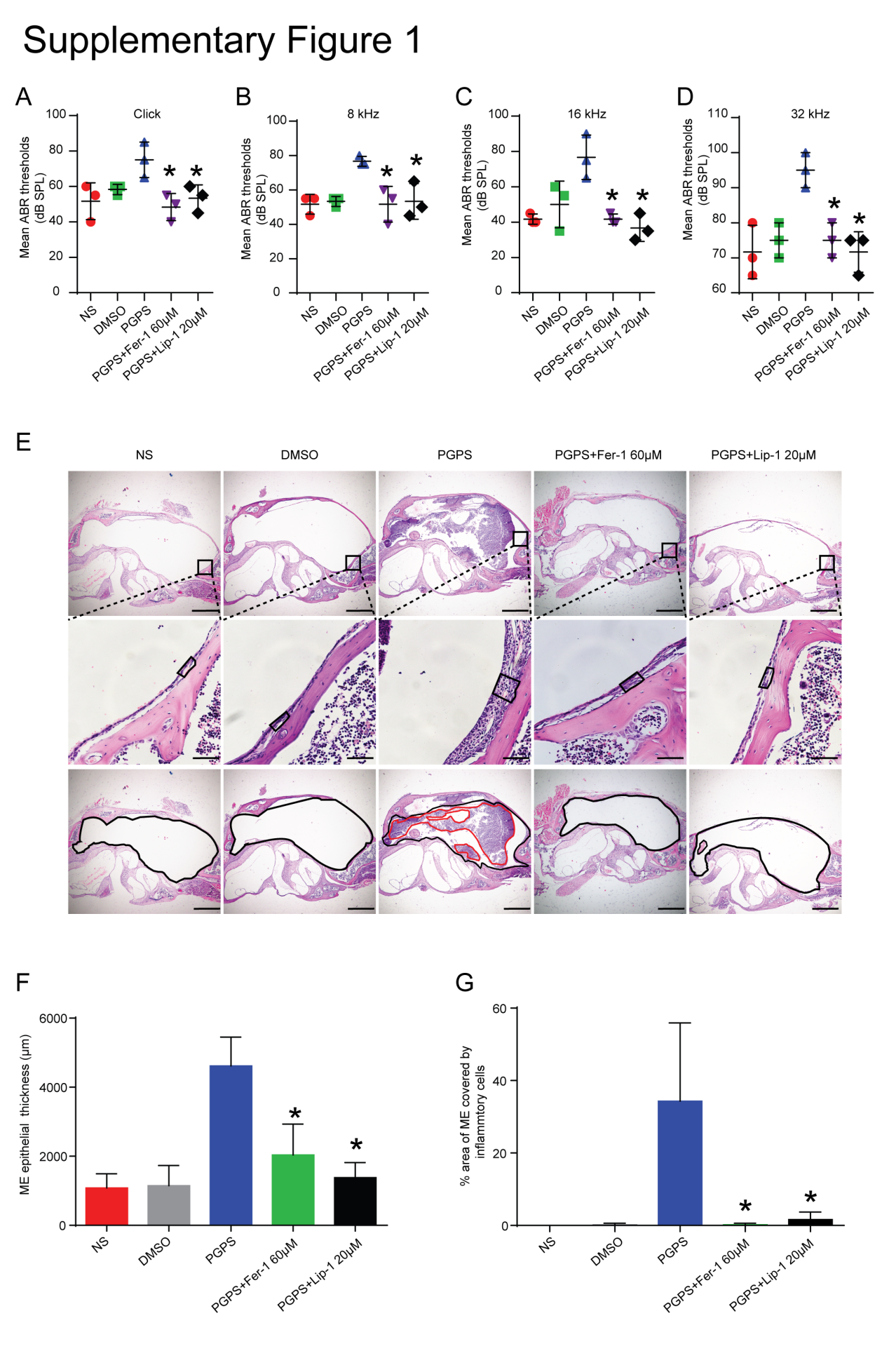


**Supplementary Figure 1 Lip-1 and Fer-1 improves the ABR threshold in mice with PGPS-induced OM and reduces inflammation of OM.** Comparison of the average ABR thresholds of mice in the saline, DMSO, PGPS, Fer-1 and Lip-1 groups for clicks of (A) 8 kHz(B), 16 kHz(C), and 32 kHz(D). The data is shown as mean ± SD **P* < 0.05, n=3. (E) H&E staining of meddle ear cavity to confirm the inflammation effect after injected different drugs The upper and lower scale bar: 500 μm. The middle scale bar: 50 μm (F) The epithelial thickness in the middle ear cavity. (G) The inflammatory cells areas in the middle ear cavity. Inflammatory cells area and middle ear epithelial thickness were analyzed with Image J. The data is shown as mean ± SD. **P* < 0.05, n=3.


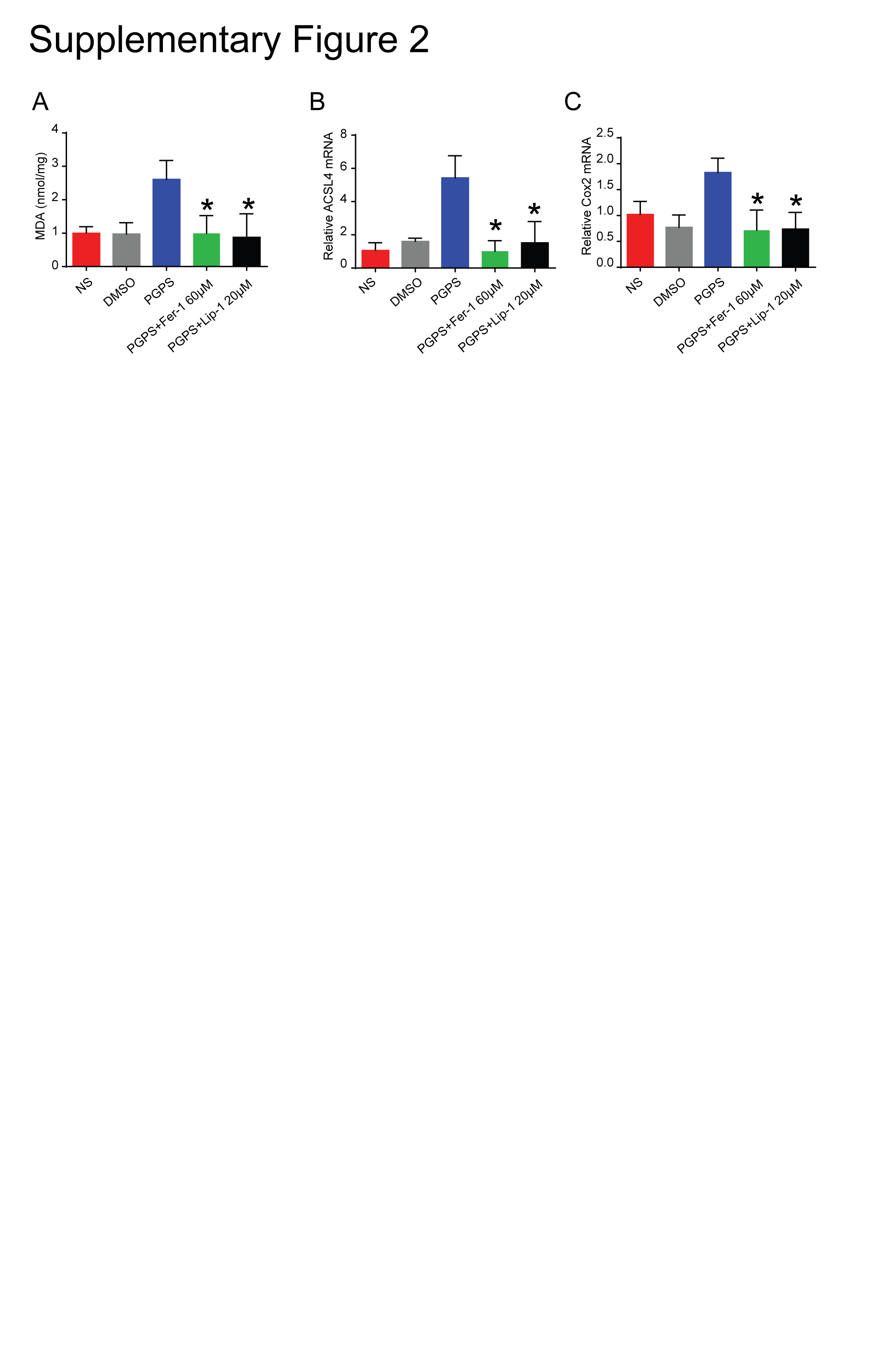


**Supplementary Figure 2 Lip-1 and Fer-1 inhibit ferroptosis in OM.** (A) Activity of amounts of MDA. (B, C) Expression of ACSL4 and Cox2 was measured by quantitative RT-PCR. The data is shown as mean ± SD. **P* < 0.05, n=3.

**Supplementary Table 1.**

| Supplementary Table 1. Sequences of primers | |
| --- | --- |
| Primers | Sequence |
| GPX4 | F:5'-GCAACCAGTTTGGGAGGCAGGAG-3' |
|  | R:5'-CCTCCATGGGACCATAGCGCTTC-3' |
| ACSL4 | F:5′-CCGACCTAAGGGAGTGATGA-3′ |
|  | R:5′-CCTGCAGCCATAGGTAAAGC-3′ |
| Cox2 | F:5’-GGGAGTCTGGAACATTGTGAA-3’ |
|  | R:5’-GTGCACATTGTAAGTAGGTGGACT-3’ |
| GAPDH | F:5’-AGGTCGGTGTGAACGGATTTG-3’ |
|  | R:5’-TGTAGACCATGTAGTTGAGGTCA-3’ |
