# Supplementary figures and images for: Ferroptosis is involved in PGPS-induced otitis media in C57BL/6 mice
Source: Cell Death Discov. 2022 Apr 21;8:217. doi: 10.1038/s41420-022-01025-1 (PMC9023543; doi:10.1038/s41420-022-01025-1)

**Figure 7G**

GPX4

**
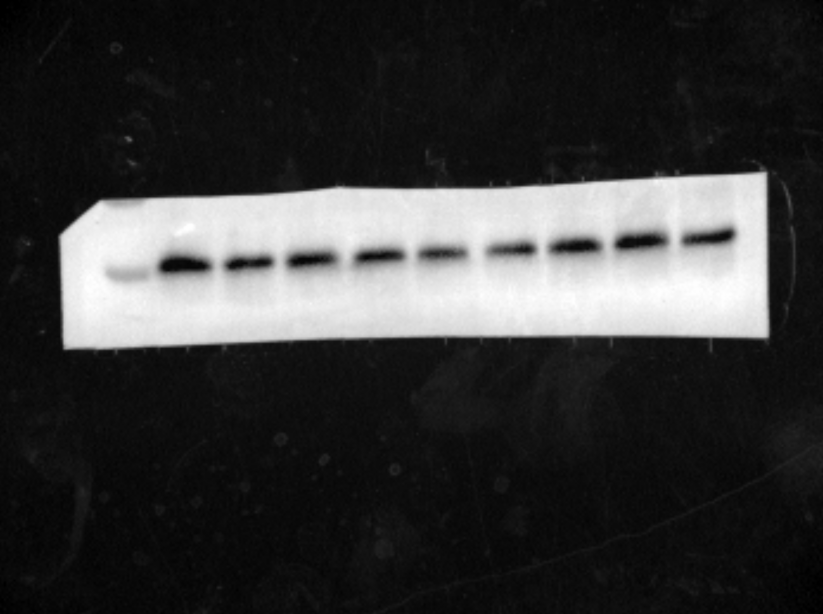
**

β-actin

**
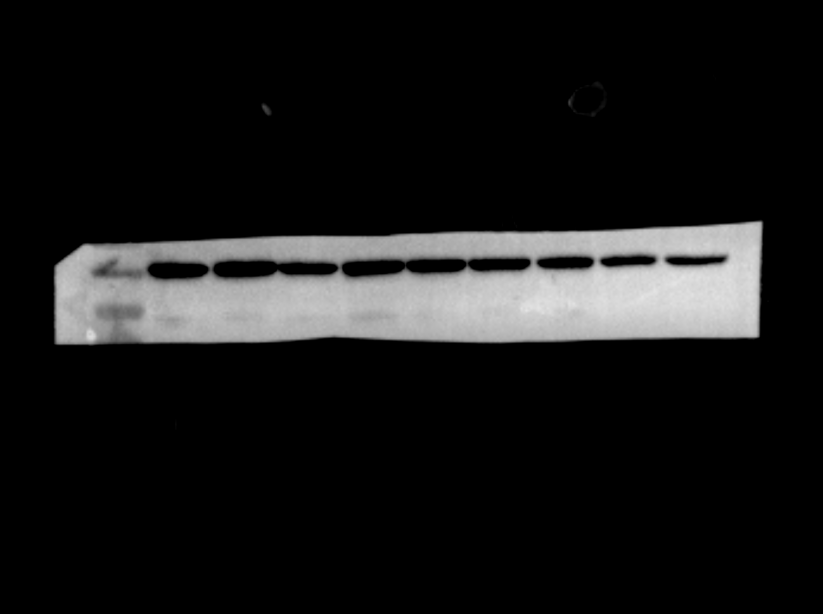
**

Cox2

**
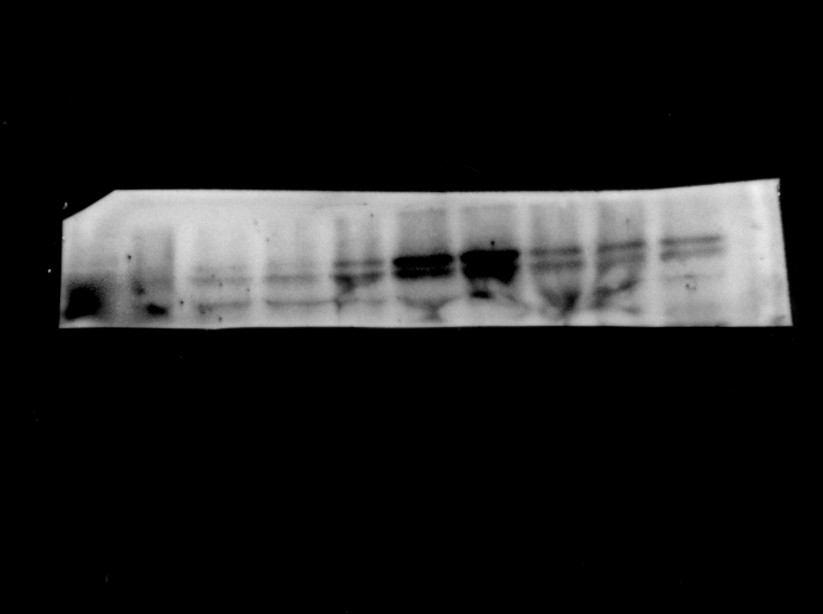
**

ACSL4


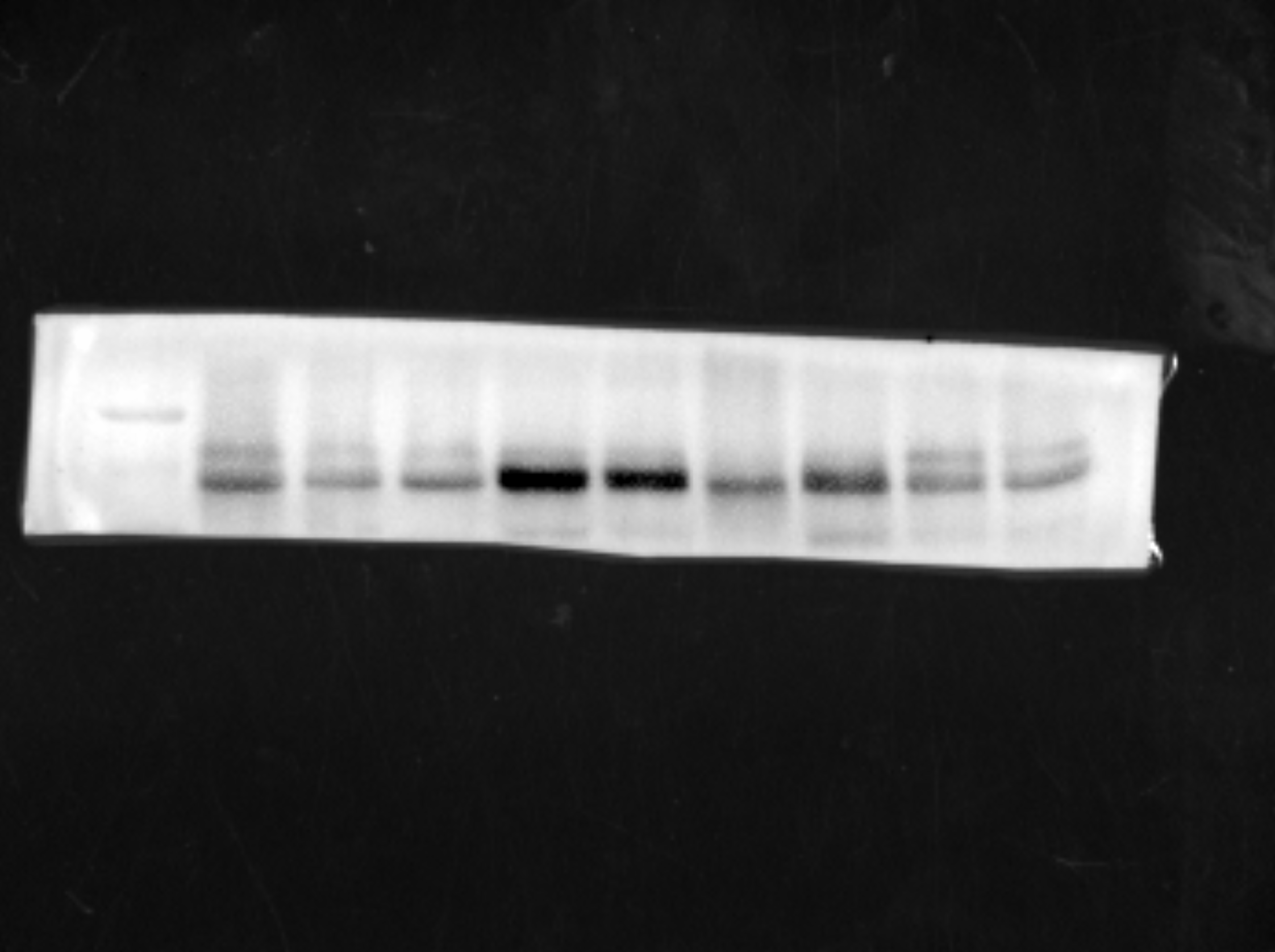

Supplement: Supplementary file 2 — supplementary material [file 41420_2022_1025_MOESM2_ESM.docx]
